# Percutaneous balloon compression for trigeminal neuralgia: experience and surgical techniques from a single institution

**DOI:** 10.1007/s13760-023-02310-1

**Published:** 2023-06-23

**Authors:** Shengze Deng, Jilai Luo, Minfang Lai, Wuyang Yang, Wenping Feng, Jinyou Ouyang, Jianguo Kuang

**Affiliations:** 1 Department of Neurosurgery, The First Affiliated Hospital of Nanchang University, Nanchang University, Nanchang, Jiangxi Province China; 2 Department of Neurosurgery, The Affiliated Hospital of Jinggangshan University, Jinggangshan University, Jian, Jiangxi Province China; 3 Department of Pharmacy, The First Affiliated Hospital of Nanchang University, Nanchang University, Nanchang, Jiangxi Province China; 4 Department of Neurosurgery, Johns Hopkins School of Medicine, Baltimore, USA

**Keywords:** Primary trigeminal neuralgia, Percutaneous balloon compression, Trigeminal ganglion

## Abstract

**Objective:**

The treatment experience and the technical skill with percutaneous balloon compression (PBC) for treatment of primary trigeminal neuralgia (TN) were summarised in a single institution.

**Methods:**

This is a retrospective review including consecutive patients with typical symptoms of uni-lateral primary TN who underwent PBC from June 2020 to September 2021 in our institution. We excluded secondary aetiologies of TN. Patient demographics, surgical techniques and outcomes were reviewed. All included patients were initially managed with carbamazepine before PBC.

**Results:**

A total of 70 patients were included. The mean length of follow-up was 10.6 months. Sixty-nine (98.6%) were successfully treated, and only one patient failed due to particularly narrow foramen ovale. Amongst successfully treated patients, 68 (97.1%) had immediate pain relief, with one having delayed relief. Sixty-eight patients (97.1%) had immediate facial numbness post-operatively and one (1.4%) presented delayed numbness 7 days after surgery. In the last follow-up, regarding facial numbness, 22 (31.9%) patients had complete resolution, whilst 46 (67.6%) had different degrees of benefit. Forty-nine (71.0%) patients developed masseter muscle weakness with recovery at 3-month follow-up. No anaesthesia dolorosa, keratitis, intracranial infection or death occurred in this study.

**Conclusion:**

PBC for treatment of TN has quick and effective result, and could be safely performed under general anaesthesia without discomfort to the patient. The common postoperative complications are facial numbness and masseter muscle weakness, with most being improved or recovered at follow-up.

## Introduction

Primary trigeminal neuralgia is a recurrent paroxysmal severe neuralgia in the distribution area of the trigeminal nerve, which characterises as sharp, lancinating or electric in quality with acute onset. Inspired by the concepts described by Taarnhφj [1] and Shelden [2], Mullan and Lichtor [3] creatively proposed the procedure of percutaneous balloon compression (PBC) on the trigeminal ganglion, that is, under X-ray guidance, the balloon catheter is penetrated into Meckel's cave through the foramen ovale, and the semilunar ganglion is compressed by balloon inflation to achieve lesioning of the trigeminal nerve for treatment of trigeminal neuralgia. Since the report of their experiences in 1983, this technology has been globally accepted and practised, and has been repeatedly verified and recognised [4–12]. In this study, we retrospectively summarised the clinical data of 70 patients with primary trigeminal neuralgia treated with PBC in our institution, with a focus on demonstrating our experience on surgical techniques to achieve successful treatment.

## Methods

### Study cohort and outcome definition

We enrolled consecutive patients evaluated in our department who underwent PBC of the trigeminal ganglion for typical symptoms of uni-lateral trigeminal neuralgia (TN) from June 2020 to September 2021. Patient medical history and symptoms were thoroughly assessed for accurate diagnosis of TN, excluding other aetiologies of facial pain resembling TN. Specific magnetic resonance imaging (MRI) scans focussed on trigeminal nerve were performed for all patients to rule out tumoral or vascular lesions in the cerebellopontine angle (CPA), or multiple sclerosis in brainstem. We also carefully examined if there was definite neurovascular conflict on trigeminal nerve by MRI. Carbamazepine, a first-line anticonvulsant medication for TN, was recommended initially for enrolled patients before PBC. Of note, the pain relief effect of carbamazepine also affirmed the diagnosis of TN. When the efficacy of carbamazepine plateaued with dose increase, or patients elected for non-conservative therapy including microvascular decompression (MVD) and neuro-ablative surgical treatments, such as stereotactic radiosurgery, radiofrequency thermo-coagulation, glycerol rhizotomy and PBC, we then recommended PBC as alternative management especially for patients who declined craniotomy, patients without MRI evidence of neurovascular conflict or those who failed other surgical options according to the guideline of European Academy of Neurology on trigeminal neuralgia [13]. We followed up all enrolled patients post-operatively and documented outcomes in detail. Barrow Neurological Institute (BNI) pain intensity score was applied to assess the level of pain post-operatively. It could be divided into five classes. Class I indicated a pain-free outcome (no pain and no medication); Class II patients had occasional pain but without taking any medication; Class III showed some pain and could be controlled with medication; Class IV patients had some reduction in their pain but not adequately controlled with medication; Class V indicated severe pain or no pain relief. Immediate failure was defined as postoperative TN without facial anaesthesia requiring continued medication for pain control. Recurrence was defined as resolution of TN for at least 3 months after PBC surgery followed by eventual relapse of ipsilateral TN with or without need for medication.

### Equipment and materials

Disposable minimally invasive balloon catheter kit for brain surgery (Shenzhen Qingyuan Medical Equipment Co., Ltd. China), 14-gauge cannula with blunt obturator (Shenzhen Qingyuan Medical Equipment Co., Ltd. China), Iodixanol injection (Jiangsu Hengrui Pharmaceutical Co., Ltd. China), Medical angiography X-ray system (UNIQ FD20/15, Philips Medical Systems Nederland B.V.).

### Operative techniques

The classical surgery (MVD) is always the first choice for patients especially those with neurovascular contact manifested by MRI. As mentioned before, PBC is recommended for patients who declined craniotomy, those without MRI evidence of neurovascular conflict or those who failed other surgical options. Patients have the will to decide the treatment after considering all the pros and cons of surgeries. After obtaining the agreement of PBC operation, we begin to perform the following procedure.

All patients underwent general anaesthesia. Patients were placed in supine position with slight extension of the neck. The skin insertion site was marked 2.5 cm lateral to the corner of the mouth, and a trajectory was set towards a point in line with the medial pupillary line and 3 cm anterior to the external auditory canal along the zygomatic arch. Under the fluoroscopy guidance, a 14-gauge cannula with blunt obturator was inserted and advanced to the external opening of foramen ovale. Once the cannula engaged the foramen ovale, the blunt obturator was removed, and a straight guiding stylet was inserted. The planum sphenoidale and posterior clinoid on the fluoroscopic images were superimposed to give the best view of the sella turcica. A lateral view was obtained to ensure proper trajectory. The guiding stylet was then removed, and a number 4 Fogarty deflated balloon was placed in the same place as the guiding stylet. A tentative inflation was made to prejudge the position and shape of the balloon. If the balloon was in undesired position or improper inflated shape, the balloon was immediately deflated and withdrawn, and the cannula repositioned until the ideal “pear shape” was achieved. The inflation volume of the balloon was between 0.3 and 0.9 ml, and the average time of semilunar ganglion compression was 2–3 min. Subsequently, the balloon was deflated, the cannula was withdrawn, and the puncture point was compressed for at least 5 min to prevent subcutaneous hematoma.

## Results

### Presenting manifestations

Based on the inclusion criteria, we enrolled 70 patients with idiopathic TN. The population consisted of 35 males and 35 females, ranging in age from 45 to 91 years, with an average of 66.4 years. Detailed information including age distribution, lesion laterality, affected trigeminal division and prior surgical treatments are shown in Table 1. The pain affected the first division (V1) of the trigeminal nerve alone accounted for 5.7% of all patients, whilst V1 distribution combined with other divisions was in another 20%. The duration of symptoms before surgery was 0.2–29 years with mean of 6.2 years.

**Table 1:** Clinical summary of 70 patients treated by percutaneous balloon compression

Variables	Patients (*N* = 70)
Sex	
Male	35 (50.0%)
Female	35 (50.0%)
Age (years)	
≤ 65	35 (50.0%)
> 65	35 (50.0%)
Lesion side	
Left	33 (47.1%)
Right	37 (52.9%)
Affected trigeminal division	
V1	4 (5.7%)
V2	21 (30.0%)
V3	9 (12.9%)
V1 + V2	11 (15.7%)
V2 + V3	22 (31.3%)
V1 + V2 + V3	3 (4.3%)
Allergy to carbamazepine	1 (1.4%)
Prior surgical treatment	31 (44.3%)
Microvascular decompression	16 (22.9%)
Radiofrequency thermo-coagulation	7 (10.0%)
Gamma knife	3 (4.3%)
Peripheral nerve blocks	5 (7.1%)

### Treatment outcome

**Fig. 1:**
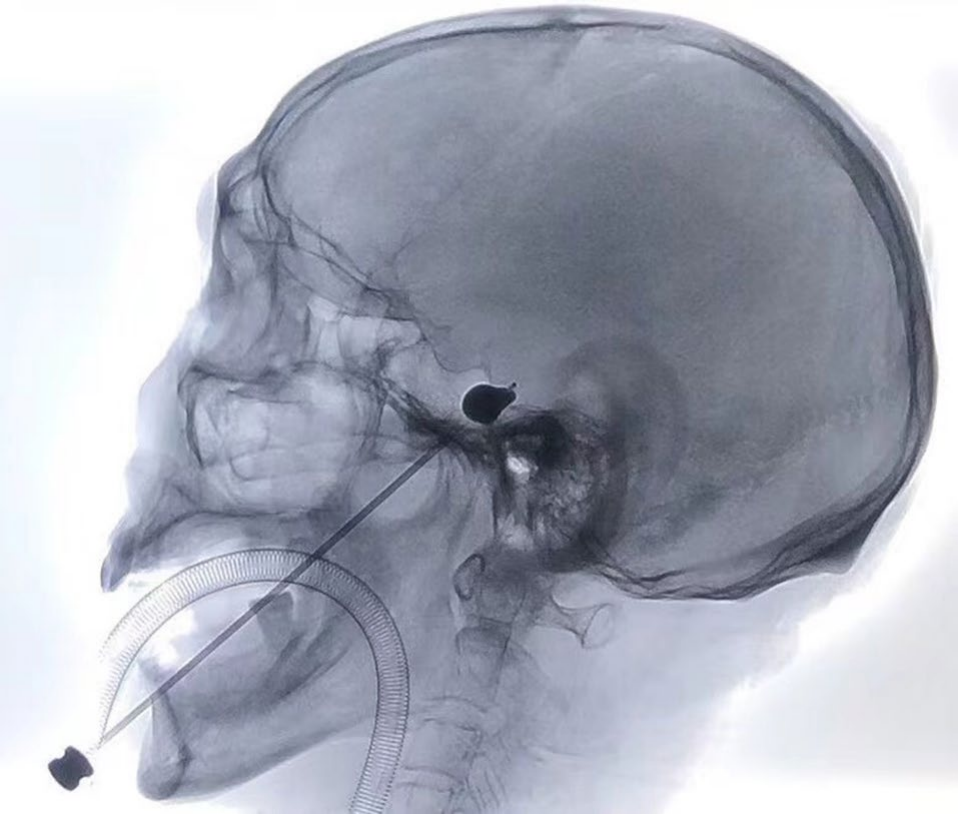
The ideal pear-shaped balloon was confirmed on lateral radiography

One patient’s foramen ovale could not be accessed because of particularly small size. Therefore, there was one patient without improvement after surgery (1.4%). Of the successfully treated patients, all 69 patients except one (who had delayed pain-relief 7 days after operation) experienced immediate relief from TN (97.1%). Amongst all the successful attempts, 63 patients (90.0%) had typical “pear-shaped” balloon (Fig. 1), whilst four had atypical “pear-shaped” balloon (5.7%), one hourglass-shaped (1.4%) and one oval-shaped (1.4%). We utilised BNI pain intensity score to assess the degree of pain relief after surgery as mentioned in Methods section. Amongst all 70 patients, 67 (95.7%) of them achieved complete pain relief without medication (Class I); 2 patients (2.9%) underwent occasional pain requiring no medication (Class II) and only 1 patient (1.4%) failed to obtain pain relief (Class V) post-operatively. Intraoperatively, 61 patients (87.1%) had trigeminal cardiac reflex with bradycardia (heart rate lower than 60 bpm) and hypertension (blood pressure elevated over 140/90 mmHg) who were administered atropine (0.5 mg) and uradil (25 mg) intravenously, whilst 6 (9.8%) of them encountered extremely transient sudden cardiac arrest when the foramen ovale was punctured. These phenomena last no longer than 10 s with spontaneous return of baseline heart rate of patients, or when the manipulation was stopped and/or atropine (0.5 mg) was given intravenously. The balloon volume was usually 0.4–0.6 ml (81.43%) and occasionally 0.9 ml of contrast medium inflated into the balloon to achieve the ideal pear shape. In early phases when we started this technique, the compression time of the trigeminal ganglion was 3 min or longer (37.1%), with some patients requiring even longer at 5–6 min (7.1%). As the technique matured, we decreased the compression time to 2 min in most patients (45.7%) or 90 s in selected few (15.7%). Details are illustrated in Table 2. Postoperatively, 68 patients developed immediate mild to moderate ipsilateral facial anaesthesia (97.1%), which was well-tolerated and gradually resolved. One patient (achieved a typical “pear-shape” balloon smoothly during surgery) who had delayed pain relief as mentioned above suffered moderate, ipsilateral hypoesthesia 7 days after surgery, and progressed into severe hypoesthesia in 3 months and maintained severe ipsilateral facial hypoesthesia at last follow-up (12 months after surgery) without improvement. A continuous follow-up of this patient is scheduled. Follow-up time varied from 5 to 18 months, with average of 10.6 months. At last follow-up, 65.7% of patients had persistent mild to moderate decreased sensation of trigeminal divisions but not affecting their daily life. Of note, 22 (31.4%) patients reported complete resolution of facial numbness. The strength of the ipsilateral masseter muscle was decreased in 49 patients (70.0%). Forty-eight (97.9%) recovered within 3 months after PBC, whilst 1 had atrophy of the masseter muscle with some limitation in mandibular activity. Twenty-four patients (34.3%) developed herpes simplex around ipsilateral lip 3 days post-operatively, symptoms persisted for about one week and recovered without anti-viral medication. One patient (1.4%) suffered transient diplopia and resolved within 3 months. There were no anaesthesia dolorosa, keratitis, intracranial infection or death in this study (Table 3). Only 2 patients had pain recurrence at the last follow-up.

**Table 2:** Operative details of 70 patients treated by PBC

Treatment variables	Patients (*N* = 70)
Puncture	
Failed	1 (1.4%)
Successful attempts	69 (98.6%)
Balloon shape	
Typical pear-shaped	63 (90.0%)
Atypical pear-shaped	4 (5.7%)
Hourglass	1 (1.4%)
Oval	1 (1.4%)
Balloon volume (ml)	
0.3	4 (5.7%)
0.4	19 (27.1%)
0.5	28 (40.0%)
0.6	10 (14.3%)
0.7	4 (5.7%)
0.8	2 (2.9%)
0.9	2 (2.9%)
Compression time (min)	
1.5	11 (15.7%)
2	32 (45.7%)
3	20 (28.6%)
4	1 (1.4%)
5	3 (4.3%)
6	2 (2.9%)
Trigeminal cardiac reflex	61 (87.1%)
Transient cardiac arrest	6 (8.6%)

**Table 3:** Outcome and complications of 70 Patients after PBC

Outcome	Patients (*N* = 70)
Immediate relief of pain	68 (97.1%)
Delayed relief of pain	1 (1.4%)
Postoperative BNI pain intensity score	
Class I	67 (95.7%)
Class II	2 (2.9%)
Class III	0 (0.0%)
Class IV	0 (0.0%)
Class V	1 (1.4%)
Complications	
Masseter muscle weakness	49 (70.0%)
Masseter muscle atrophy	1 (1.4%)
Herpes simplex	24 (34.3%)
Diplopia	1 (1.4%)
Anaesthesia dolorosa	0 (0.0%)
Keratitis	0 (0.0%)
Intracranial infection	0 (0.0%)
Death	0 (0.0%)
Hypoesthesia (3 months after surgery)	
Severe	1 (1.4%)
Moderate	15 (21.4%)
Mild	31 (44.3%)

## Discussion

Our study has shown that PBC provided immediate postoperative pain relief for 97.1% of TN patients. None of the patients had severe complications, such as anaesthesia dolorosa, corneal anaesthesia, intracranial infection, intracranial haemorrhage or death. Only 2 patients had pain recurrence during follow-up.

The target site of radiofrequency or glycerol rhizotomy is one or several nerve trunks of the trigeminal nerve, which injures both unmyelinated and myelinated fibres [6], whilst PBC is performed to indiscriminately compress the entire trigeminal ganglion. Animal experiments have shown that compression preferentially injured large, myelinated fibres, removing the “trigger” to the presumed ephaptic transmission of pain. Provided that unmyelinated fibres which mediated the corneal reflex were preserved, compression may be advantageous in treatment of first division pain in TN, since the corneal is innervated by small, unmyelinated fibres [6]. Moreover, this differential injury of axons and sparing of trigeminal ganglion cell bodies suggests that axonal regeneration is possible and may contribute to the recovery of motor and sensory function in patients after PBC [14], which correlated with only transient facial numbness of most of our patients.

The technical aspect of PBC is not complicated when compared with MVD. The learning curve for PBC is approximately 3–6 months for a trained neurosurgeon to reach a proficient level. In our early phase, the mean time for PBC surgery lasted about 2 to 3 h or sometimes even longer. After 3–6 month practice, we gradually felt that the manipulation of foramen ovale puncture is getting better and better and the number of times to adjust the trajectory of puncture became less and less. The operative technic matured obviously. As a result, the procedure time decreased into less than one hour or even 30 min. Also in the early phase of our study, the compression time was intentionally prolonged to ensure optimal effectiveness of the surgery. The main factor for decision of extending the compression time is the shape of balloon. During our early phase of this study, judgement of the ideal “pear-shaped” balloon was with difficulty. In order to achieve good results, we often tried several times on adjusting the puncture trajectory in order to place the cannula in an ideal position and harvest a useful "pear-shape" balloon ultimately. So the cumulative compression time was prolonged. Therefore, mean compression for most patients was more than 3 min, whilst the longest case was divided into two sections, with a total of 6 min. As the operative technic matured, we recognised that pain relief was not proportionally related to compression time; on the contrary, prolonged compression will result in severe and long-lasting facial numbness after surgery [4, 10, 15]. Therefore, we reduced the compression time to 100–120 s, and achieved similar effectiveness for pain control whilst ensuring a low postoperative complication rate.

**Fig. 2:**
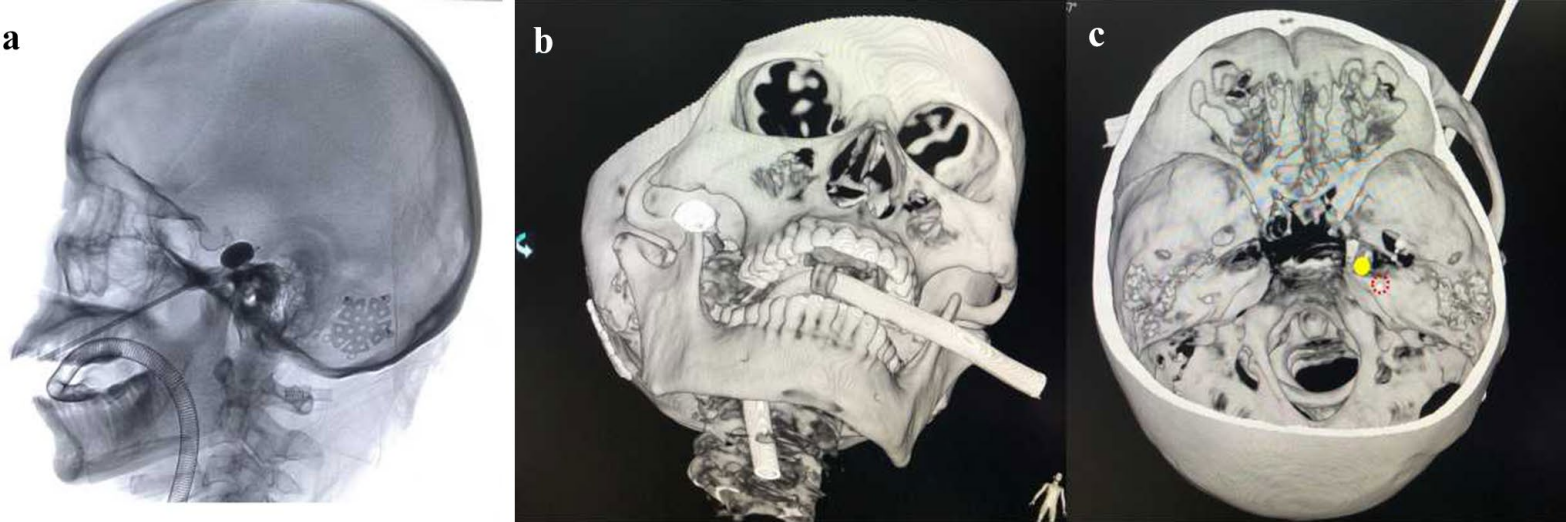
Inaccurate position of balloon catheter before adjustment. **a** The balloon shape is cylindrical on lateral radiography. **b** The tip of puncture cannula is projected on the lateral parts of the external opening of foramen ovale from external appearance of skull base in 3D reconstructed CT imaging. **c** The tip of balloon catheter (red dotted circle) is deviated from the trigeminal porus (yellow solid dot) from internal appearance of skull base in 3D reconstructed CT imaging (color figure online)

**Fig. 3:**
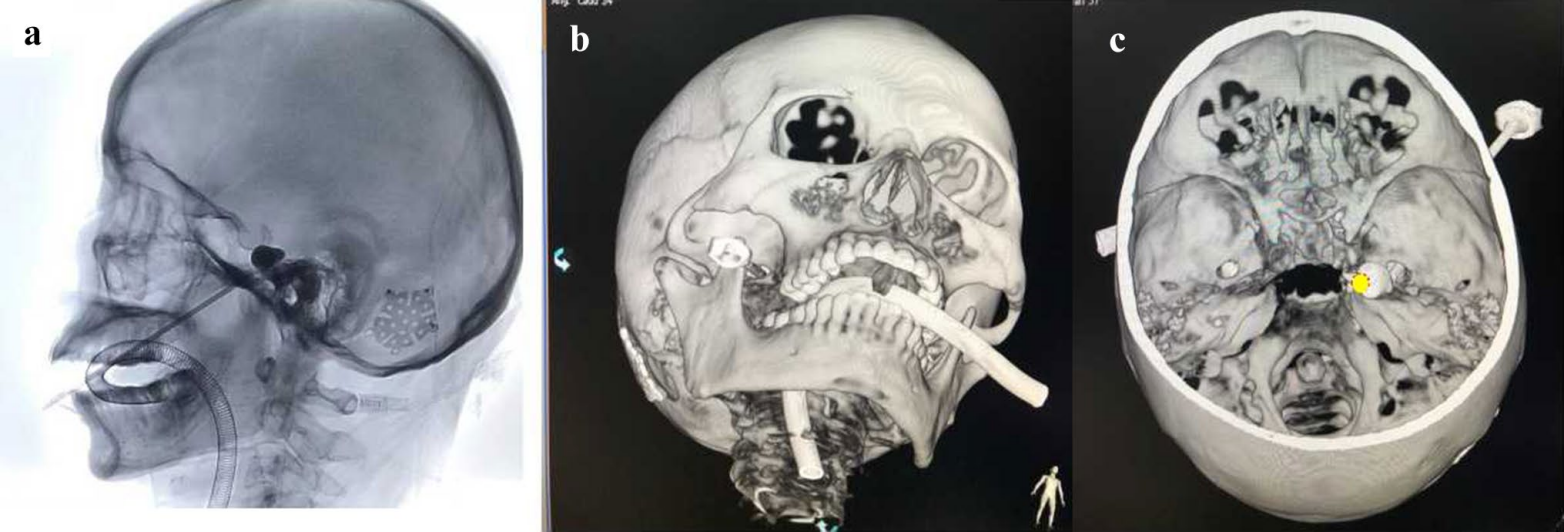
Accurate position of balloon catheter after adjustment. **a** Ideal pear-shaped balloon on lateral radiography. **b** The tip of puncture cannula is projected on the lower medial parts of the external opening of foramen ovale from external appearance of skull base in 3D reconstructed CT imaging. **c** The tip of balloon catheter (red dotted circle) is almost overlapped with the trigeminal porus (yellow solid dot) from internal appearance of skull base in 3D reconstructed CT imaging (color figure online)

The golden standard for maximal curative effect of PBC surgery is the formation of ideal “pear-shaped” balloon after inflation on lateral radiography [3]. The authors of this paper would like to share some operative pearls developed in our department to obtain a successful “pear-shape” of the balloon. When the puncture cannula is placed at the external opening of foramen ovale or between the internal and external openings of foramen ovale, the tail of the cannula will sustain a tactile feedback similar to that of a lumbar puncture. Additionally, engagement of the foramen occasionally triggers contraction of the masseter and pterygoid muscles, as well as sudden invagination of the skin at the puncture site. These signs indicate successful puncture of the external opening of foramen ovale, but not necessarily predict a smooth entrance of the balloon catheter into Meckel’s cave. In some cases where the balloon did not form ideal “pear-shape”, we would deflate the balloon immediately, maintain the position of the balloon catheter at the foramen, and obtain a 3-dimensional (3D) CT imaging. From the 3D images, external view of the skull base was reviewed first, which we have found that in most of the cases, the tips were projected on the upper or lower lateral parts of the external opening of foramen. Next, a review of internal view of the skull base was performed, which revealed most were deviated from the trigeminal porus (Fig. 2), suggesting that the balloon catheter was not in the Meckel’s cave. Adjustment was made based on 3D CT images and a subsequent 3D CT at final position of the balloon catheter demonstrated optimal positioning at the trigeminal porus (Fig. 3). It is to our experience that a higher probability of success can be reached by adjusting the trajectory towards the lower medial direction when failed to reach the Meckel’s cave. In the early phase of PBC, we recommend intraoperative 3D CT imaging for shortened operation time and improve success rate. Neuro-navigation-assisted or even robot-assisted PBC was recommended in some articles especially for those inexperienced neurosurgeons [16–18]. These assisted systems may help shorten the learning curve of beginners and may be useful in anatomic variation cases with difficulty to access the foramen ovale. Compared with non-navigation group, the first puncture success rate of the navigation group was elevated obviously as they reported. But the puncture success rate is actually not equal to the surgical success rate because placing the balloon catheter into Meckel's cave accurately is the key point as we mentioned above. Time-consuming preparation and registration work together with more complicated procedure and potential risk when using 3-pin head holder during the surgery may limit the application of neuro-navigation and robot-assisted devices. Anyway, these assisted systems may provide a good choice for the institutions who are willing to start the PBC surgery.

We would also like to discuss the definition of “pear-shape” and the necessity to obtain the optimal shape of the balloon. On the lateral view of intraoperative X-rays, the ganglion is located at the bottom of the contrast medium-inflated balloon. The optimal position of the balloon catheter is confirmed (located in Meckel’s cave) when a bleb of balloon emerged, resembling the “pear head”. Then the pressure at the bottom of the balloon should be strengthened to make the “pear” in a shape of “small pear head and large pear body”. This technique would guarantee the best curative effect in a relatively short compression time. The pressure of the balloon can be sensed by the tactile feedback of the resistance transmitted to the syringe, and maintaining the state of maximum resistance can ensure that the pressure at the bottom of the balloon is at maximum capacity. Conversely, Mullan et al. believed that if the pear shape appears, it is not necessary to maximally inflate the balloon, which could reduce or even prevent postoperative facial numbness [10]. In our experience, maximal balloon inflation is the key to achieve definitive effect within a short duration of time, which would reduce the degree of postoperative facial numbness. This is consistent with studies showing that in PBCs, immediate postoperative outcome is positively associated with the intensity of compression [19]. Of note, a certain degree of numbness often indicated a satisfactory operative outcome of lesioning the trigeminal nerve. The numbness was not concerning to patients and would gradually decrease or resolve during follow-up, which is consistent with other literatures [12, 20].

Intraoperative trigeminal cardiac reflex is a relatively common occurrence [21] and requires attention. In our study cohort, 61 (87.1%) patients had a significant decrease in heart rate and elevation in blood pressure. Transient cardiac arrest occurred in 6 patients, and sinus rhythm was restored after cessation of procedures and/or intravenous atropine injection. Although trigeminal cardiac reflex can be used as an secondary indicator to determine whether foramen ovale is punctured, and whether the balloon catheter is located in Meckel’s cave; however, awareness needs to be raised and early response to avoid anaesthetic and surgical risk should be initiated to prevent severe consequences. Nevertheless, none of our patients had complications from this phenomenon. Some scholars suggest that a temporary pacemaker can be routinely placed before surgery [22]. In our practice, we also agree that for elderly patients with sinus bradycardia or other arrhythmias before surgery, placement of temporary pacemaker in advance is recommended to ensure the safety of surgery.

There are limitations of this study that need to be addressed. First, this is a single institution experience with retrospective review of cases, selection and recall bias is inevitable. Next, we have not directly compared the results of our case series with rhizotomies in this study, which would be the future direction of our ongoing research effort. At last, the follow-up duration is relatively short at 10.6 months, which may not be long enough to detect recurrences of TN. Despite all these, our goal in this paper was to introduce operative techniques that may achieve satisfactory outcome of TN treatment using PBC, and we think our institutional experience will provide experience which can be disseminated, evidence and guidance for future PBC setup in other institutions.

## Conclusion

We present in this study a case series demonstrating satisfactory outcome of PBC in TN treatment. The effect of PBC can be quick and durable, and safely performed under general anaesthesia. Operative pearls of PBC including diagnosing the tip position of balloon catheter and the necessity of achieving maximal “pear-shape” were discussed in detail. We believe the experience introduced in this study can be generalised and referenced for future PBC procedures.

## Data Availability

The authors confirm that the data supporting the findings of this study are available within the article.

## References

[1] Taarnhφj P (1952). Decompression of the trigeminal root and the posterior part of the ganglion as treatment in trigeminal neuralgia. Preliminary communication. J Neurosurg.

[2] Shelden CH, Pudenz RH, Freshwater DB, Crue BL (1955). Compression rather than decompression for trigeminal neuralgia. J Neurosurg.

[3] Mullan S, Lichtor T (1983). Percutaneous microcompression of the trigeminal ganglion for trigeminal neuralgia. J Neurosurg.

[4] Abdennebi B, Mahfouf L, Nedjahi T (1997). Long-term results of percutaneous compression of the gasserian ganglion in trigeminal neuralgia (series of 200 patients). Stereotact Funct Neurosurg.

[5] Belber CJ, Rak RA (1987). Balloon compression rhizolysis in the surgical management of trigeminal neuralgia. Neurosurgery.

[6] Brown JA, Gouda JJ (1997). Percutaneous balloon compression of the trigeminal nerve. Neurosurg Clin North Am.

[7] Correa CF, Teixeira MJ (1998). Balloon compression of the Gasserian ganglion for the treatment of trigeminal neuralgia. Stereotact Funct Neurosurg.

[8] Fraioli B, Esposito V, Guidetti B, Cruccu G, Manfredi M (1989). Treatment of trigeminal neuralgia by thermocoagulation, glycerolization and percutaneous compression of the gasserian ganglion and/or retrogasserian rootlets: long-term results and therapeutic protocol. Neurosurgery.

[9] Frank F, Fabrizi AP (1989). Percutaneous surgical treatment of trigeminal neuralgia. Acta Neurochir.

[10] Lichtor T, Mullan JF (1990). A 10 year-follow-up review of percutaneous microcompression of the trigeminal ganglion. J Neurosurg.

[11] Lobato RD, Rivas JJ, Sarabia R, Lamas E (1990). Percutaneous microcompression of the gasserian ganglion for trigeminal neuralgia. J Neurosurg.

[12] Skirving DJ, Dan NG (2001). A 20-year review of percutaneous balloon compression of the trigeminal ganglion. J Neurosurg.

[13] Bendtsen L, Zakrzewska JM, Abbott J, Braschinsky M, Di Stefano G, Donnet A, Eide PK, Leal PRL, Maarbjerg S, May A, Nurmikko T, Obermann M, Jensen TS, Cruccu G (2019). European academy of neurology guideline on trigeminal neuralgia. Eur J Neurol.

[14] Preul MC, Long PB, Brown JA, Velasco ME, Weaver MT (1990). Autonomic and histopathological effects of percutaneous trigeminal ganglion compression in the rabbit. J Neurosurg.

[15] Lee ST, Chen JF (2003). Percutaneous trigeminal ganglion balloon compression for treatment of trigeminal neuralgia, part II: results related to compression duration. Surg Neurol.

[16] Dong FY, Zhan Q, Shao ZK, Gu Q, Gao XT, Zhou B, Li L, Ma YW, Wang XF, Liang YC (2022). Clinical study on the treatment of primary trigeminal neuralgia by robot-assisted percutaneous balloon compression. Front Surg.

[17] Georgiopoulos M, Ellul J, Chroni E, Constantoyannis C (2014). Minimizing technical failure of percutaneous balloon compression for trigeminal neuralgia using neuronavigation. ISRN Neurol.

[18] Aydoseli A, Akcakaya MO, Aras Y, Sabanci PA, Unal TC, Sencer A, Hepgul K, Unal OF, Barlas O, Izgi N (2015). Neuronavigation-assisted percutaneous balloon compression for the treatment of trigeminal neuralgia: the technique and short-term clinical results. Br J Neurosurg.

[19] Brown JA, Pilitsis JG (2005). Percutaneous balloon compression for the treatment of trigeminal neuralgia: results in 56 patients based on balloon compression pressure monitoring. Neurosurg Focus.

[20] Chen JF, Tu PH, Lee ST (2011). Long-term follow-up of patients treated with percutaneous balloon compression for trigeminal neuralgia in Taiwan. World Neurosurg.

[21] Brown JA, Preul MC (1988). Trigeminal depressor response during percutaneous microcompression of the trigeminal ganglion for trigeminal neuralgia. Neurosurgery.

[22] Brown JA (2009). Percutaneous balloon compression for trigeminal neuralgia. Clin Neurosurg.

